# The Pathophysiology of Farnesoid X Receptor (FXR) in the GI Tract: Inflammation, Barrier Function and Innate Immunity

**DOI:** 10.3390/cells10113206

**Published:** 2021-11-17

**Authors:** Kemp M. Anderson, Christopher P. Gayer

**Affiliations:** 1 Department of Surgery, Keck School of Medicine, University of Southern California, Los Angeles, CA 90033, USA; kemp.anderson@med.usc.edu; 2 Division of Pediatric Surgery, Childrens Hospital Los Angeles, Los Angeles, CA 90027, USA

**Keywords:** Farnesoid X Receptor, intestine, innate immunity, inflammation, fibroblast growth factor, small heterodimer protein, nuclear receptor, barrier, bile acids, gastrointestinal

## Abstract

The Farnesoid-X Receptor, FXR, is a nuclear bile acid receptor. Its originally described function is in bile acid synthesis and regulation within the liver. More recently, however, FXR has been increasingly appreciated for its breadth of function and expression across multiple organ systems, including the intestine. While FXR’s role within the liver continues to be investigated, increasing literature indicates that FXR has important roles in responding to inflammation, maintaining intestinal epithelial barrier function, and regulating immunity within the gastrointestinal (GI) tract. Given the complicated and multi-factorial nature of intestinal barrier dysfunction, it is not surprising that FXR’s role appears equally complicated and not without conflicting data in different model systems. Recent work has suggested translational applications of FXR modulation in GI pathology; however, a better understanding of FXR physiology is necessary for these treatments to gain widespread use in human disease. This review aims to discuss current scientific work on the role of FXR within the GI tract, specifically in its role in intestinal inflammation, barrier function, and immune response, while also exploring areas of controversy.

## 1. Introduction

The nuclear Farnesoid-X Receptor (FXR) was discovered in the 1990s and named due to its activation by farnesol metabolites [1]. In 1999, bile acids were discovered as the endogenous FXR ligands [2,3,4]. Upon activation, FXR forms a heterodimer with Retinoid-X receptor (RXR) to target promoters including small heterodimer protein (SHP), fibroblast growth factor 15/19 (mouse/human), and intestinal bile acid binding protein (IBABP) [5,6,7]. There are two FXR genes, α(NR1H4) and β(NR1H5), with FXRβ being a pseudogene in humans [8,9], and four isoforms expressed in a tissue-specific manner [10]. While all are referred to as FXR, the heterogeneity seen in FXR responses may be due in part to differential isoform activity [8,11].

FXR activation is achieved endogenously by most bile acids, with chenodeoxycholic acid (CDCA) binding most avidly [3]. However, development of pharmacologic FXR agonists has increased recently and include INT-747 (obeticholic acid, OCA), INT-767 (FXR and TGR5 co-agonist) [12], GW4064, Tropifexor [13], GS-9674, and EDP305 [14]. Many of these agonists are not specific to FXR and bind other non-FXR receptors, including TGR5 [13]. Developing therapeutics targeting FXR has been a recent focus [6,14,15,16,17,18].

**Figure 1:**
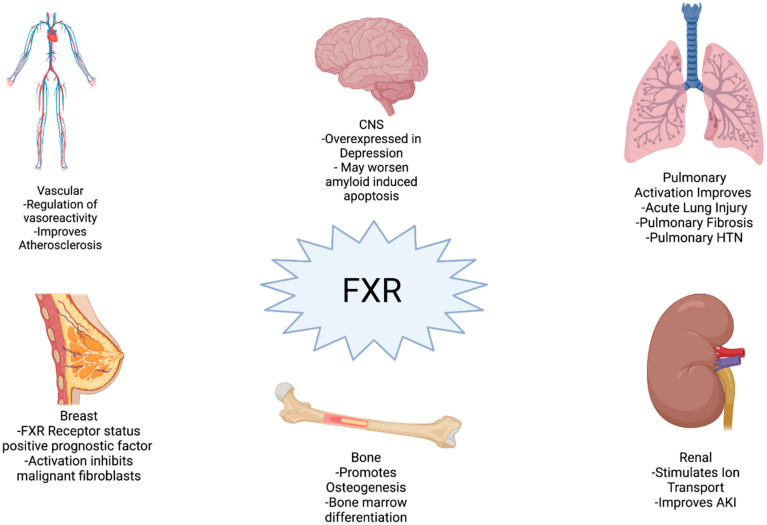
Diverse organ system effects. FXR has functions in many organ systems throughout the body outside the GI–liver axis. In many systems FXR promotes positive effects while its effects in the CNS provide counter examples of potential FXR-induced damage (created with BioRender.com, accessed on 10 October 2021) [19,21,22,23,24,25,27,34,35,36,37,38,39,40].

Indeed, FXR expression is ubiquitous, and plays a physiologic role in multiple organ systems including cardiac [19], endocrine/metabolic [20], renal [21], pulmonary [22], breast [23], vascular [24], psychiatric [25], and nervous systems [26,27] (Figure 1). The most well-described function of FXR is within the GI-liver axis, specifically its role in bile acid homeostasis. In the liver, the FXR downstream effector SHP inhibits the cytochrome p450 enzyme responsible for the rate-determining step of cholesterol transformation into bile acids. FXR also limits local uptake of bile acids in the gastrointestinal epithelium via SHP-mediated inhibition of ASBT, a bile acid transporter. This allows bile acids, via FXR, to regulate their own synthesis. The role of FXR in bile acid homeostasis are masterfully addressed in other reviews [28,29]. Other excellent reviews discuss FXR function in the liver [8,17,30], FXR ligands [8], nuclear receptor function [31], FXR in IBD [30], FXR in the gut–liver interaction [18,28], metabolic signaling [32], and therapeutics [14,33]. This review focuses on the less understood but more recently studied roles of FXR on inflammation, barrier function, and innate immunity within the GI tract.

## 2. Inflammation

The inflammatory cascade and its dysregulation provide the underlying physiological basis for many gastrointestinal diseases, including Crohn’s disease, ulcerative colitis, Clostridioides difficile colitis, necrotizing enterocolitis, and others. Gut barrier breakdown is often the ultimate cause of mortality; however, gut inflammation is frequently the initial insult. The current literature suggests that FXR plays an important role in intestinal inflammation.

### 2.1. Acute Inflammation

Generally, the literature suggests that FXR activation in acute inflammation is protective in a variety of inflammatory models. For example, in dextran sulphate sodium (DSS) colitis, FXR activation via OCA decreases local IL1β and increases systemic IL10 expression [41]. In cirrhotic rat models, FXR activation leads to a local decrease in ileal IFNγ, TNFα, MadCAM-1, and IL17 expression [42]. In murine models of ischemia-reperfusion injury (IRI), FXR activation via OCA also provides a protective effect as defined by cytokine balance. FXR activation prior to IRI maintains L-lactate, I-FABP, and LDH at control levels and blunts increases in IL6, IL1β, and IFNγ. In this model, FXR limits autophagic influx by inhibiting transcription of proteins that are required for recruitment [43]. Additionally, activation of FXR decreases the activity of diamine oxidase (DAO), an enzyme inversely correlated with small bowel integrity, and increases activity of cystathionine-γ-lyase (CSE), an enzyme responsible for production of H_2_S that improves response to oxidative stress [44]. H_2_S is important for maintenance of gastrointestinal mucosa [45] and has been previously described to be a target of FXR activation [46]. These results suggest that FXR activation blunts intestinal inflammation in pathologic states.

However, there are conflicting reports within the literature that challenge this role of FXR. For example, cirrhotic rats, which are known to have intestinal inflammation, treated with OCA showed decreased anti-inflammatory IL-10 expression relative to control mice, suggesting that there are disease- or model-specific differences in FXR-regulated cytokine expression that are not understood [42]. In an oxygen-glucose deprivation and reperfusion model, FXR activation via OCA was shown to enhance cell viability in a dose-dependent manner and to decrease pro-inflammatory cytokines NFκB, TNFα, and IL-6 [44]. However, a nearly opposite effect was seen in Caco2 cells treated with the native FXR agonist CDCA [47]. Others have shown that FXR activation via CDCA resulted in significantly increased pro-inflammatory IL8, IL6, TNFα, and vascular endothelial growth factor. Inhibition of FXR in Caco2 cells via Z-guggulsterone blocked CDCA-induced IL8, IL6 and TNFα release and blocked increases in IL8 and IL6 mRNA expression [47]. These conflicting results may be attributed to specific animal models or cell lines differences but point to a complex interaction between FXR and the intestine.

Acute inflammatory cytokines including TNFα decrease FXR expression by decreasing DNA binding activity at the FXR response element [48], suggesting that not only does FXR modulate inflammatory signaling, but inflammatory modulators can also affect FXR expression. This feedback loop is similar to FXRs control over bile acid metabolism [28]. Pro-inflammatory cytokines downregulate FXR expression in the setting of lipopolysaccharide (LPS)-mediated inflammation [48] and FXR is downregulated in DSS colitis intestine [49]. Interestingly, Gadaleta et al. demonstrated the same results ex vivo in human colon carcinoma cells when they showed TNFα, IL1β, and NFκB inhibit FXR activity. They demonstrated that NFκB subunits inhibit downstream FXR transcriptional activity by binding directly to FXR [49]. Similar downregulation of the FXR-FGF15 axis has been observed in other models of murine colitis [50], leading to inhibition of FXR downstream targets [48,50]. Intestinal FXR and FGF19 expression is also lower in premature pigs, another physiologic model of inflammatory stress [51]. Thus, while FXR seems to be protective when activated in the setting of acute inflammation, acute phase responses themselves have a negative feedback effect on FXR expression.

### 2.2. Chronic Inflammation

In chronic inflammatory settings, FXR activation also plays an important and similar role, although the conflicting reports appear greater. While chronically maintained on a highly inflammatory diet, fish models develop a pro-inflammatory phenotype with increases in TNFα, COX-2, IL1β, and IL6 and decreases in IL10 [52]. They also have decreased intestinal FXR gene expression, consistent with work described above [48]. In these fish, FXR activation via dietary CDCA abrogated pro-inflammatory changes to the cytokine profile [52]. In contrast, mice that were chronically fed pro-inflammatory “western-type” diets heavy in deoxycholic acid (DCA), another strong physiologic FXR agonist, showed a decrease in ileal FXR and FGF15 expression. This exposure led to increased expression of ileal and colonic IL1β, IL6, and TNFα [53], opposite to what was expected. While these results were attributed to an underlying DCA-induced dysbiosis, it does raise questions regarding the potential for differential effects of FXR on chronic versus acute inflammatory settings.

Genetic knockout of FXR in animals leads to a baseline pro-inflammatory profile. Global FXR KO mice have increased expression of pro-inflammatory IL6 [54], TNFα, IL1β, IFNγ, TGF1, and TIMP1 [55]. Additionally, when exposed to pro-inflammatory insults, FXR KO mice produce significantly higher amounts of IFNγ, TNFα, IL1β [56], and diamine oxidase [44]. They may also be less responsive to oxidative stress, producing less CSE in response to injury [44]. The baseline phenotypic differences in FXR KO also leads to structural changes suggestive of a chronic, pro-inflammatory state, including chronic cellular infiltration of the colonic lamina propria and enhanced collagen deposition within the intestinal wall. Effects such as enhanced extracellular-matrix protein and smooth muscle actin expression, which are associated with chronic inflammation of other systems [55], are seen within 14–15 weeks of life. A summary of described FXR effects in these different inflammatory models is shown in Table 1.

Since most evidence suggests decreasing pro-inflammatory cytokines with FXR activation, it stands to reason that manipulating downstream FXR targets would be beneficial. In DSS colitis, treatment with the FGF15 analogue M52 decreases IL1β, IL6, and TNFα [57]. These findings are consistent with observations that murine colitis is attenuated following treatment with recombinant FGF15 [50]. Interestingly, these effects may still be FXR-dependent, as DSS was not improved in FXR KO mice treated with M52 [57]. The reason for these results is unclear. Perhaps the described linear FXR-FGF15 pathway is more complex, or perhaps there are FXR-dependent effects that are FGF15-independent.

This connection between FXR and inflammation is made more confusing in DSS and IL10 KO colitis. Here, there is seemingly no activation of FXR-FGF15 pathway at baseline, yet these mice have higher expression of apical sodium-dependent bile acid transporter, which is negatively regulated by FXR. In fact, IL10 KO mice have decreased FXR expression [58]. This suggests a deleterious FXR pathway inhibition in these disease models.

FXR signaling appears linked to the intestinal peroxisome proliferator-activated receptor (PPARα)-UDP-glucuronosyltransferases (UGTs) axis. PPARα-UGT is important for bile acid homeostasis, as glucuronidation alters bile acids for easier transport and excretion. The PPARα-UGT is upregulated in murine colitis, leading to a decrease in the total bile acid pool, which leads to decreased FXR activation. Thus, upregulation of PPARα-UGT leads to decreased FXR activity. Interestingly, in the setting of DSS, PPARα KO mice maintain baseline FXR expression and experience attenuated injury relative to WT mice. Conversely, mice that are exposed to activators of PPARα prior to DSS exposure show decreased FXR expression and have worse survival [50]. These data again reflect the seemingly variable FXR-related responses to inflammation within the GI tract.

### 2.3. Glucocorticoid Receptor Interactions

**Figure 2:**
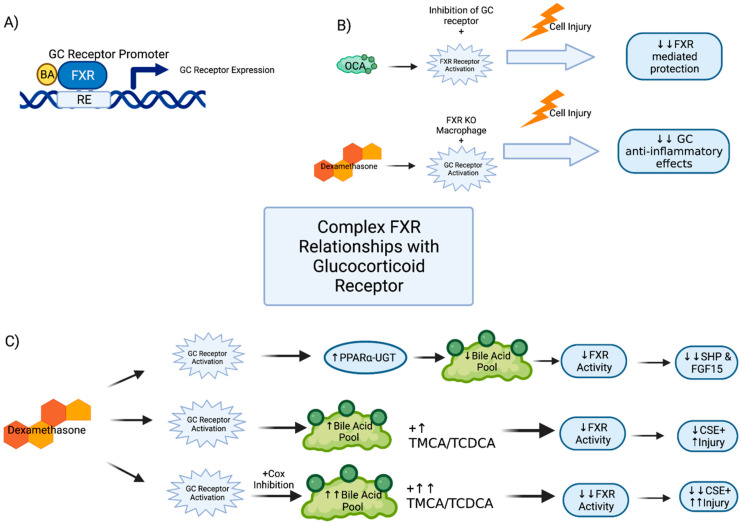
FXR relationship to glucocorticoid receptor function. There are complicated interactions between FXR and GC receptor in both their respective and synergistic responses to cellular insult. (**A**) Transcriptional response elements within the promoter sequence for glucocorticoid receptor RE—response element. (**B**) Synergistic anti-inflammatory relationship between FXR and GC receptor function such that diminishing activity of one affects functional response to injury of the other. OCA—obeticholic acid (**C**) GC receptor activation decreases functional effects of FXR; however, different mechanisms for this effect have been proposed. Additional relationship between FXR, GC receptor and COX activity have also been observed (CSE = cystathione-γ-lyase; SHP—small heterodimer protein, FGF-fibroblast growth factor) (Created with BioRender.com accessed on 10 October 2021).

The role of FXR has been investigated in other well-defined gastrointestinal inflammatory pathways, including the glucocorticoid receptors (GR) pathway, which bears similarity to FXR as a nuclear receptor, and the prostaglandin-cyclooxygenases (COX) pathway. Both GR and COX pathways are routinely manipulated clinically. It is well known that non-steroidal anti-inflammatory (NSAID) therapies can lead to gastrointestinal injury, and glucocorticoid anti-inflammatory therapy has many off-target effects [59]. Given the role of FXR in gut inflammation, one would expect FXR to interact with these pathways. In fact, it has been shown that FXR KO mice have reduced expression of GR relative to WT and are less responsive to glucocorticoid therapy in inflammation [56]. A similar differential response to glucocorticoid therapy is observed in immune cells. When treating WT pro-inflammatory (M1)-polarized macrophages with dexamethasone, a dose-dependent decrease in pro-inflammatory cytokines TNFα, IL1β, and IFNγ is seen. However, in M1 macrophages from FXR KO mice, a 10-fold higher dose of dexamethasone is required to observe similar cytokine reductions. Furthermore, co-administration of a GR antagonist lead to the abrogation of anti-inflammatory effects typically seen with the FXR agonist OCA [56]. In genomic analysis of mice, an FXR-response element was found in the murine GR promoter [56], which suggests that some level of FXR activity may be mediated by the glucocorticoid pathway (Figure 2A,B).

These data suggest a complex relationship between FXR and GR. This is supported by data using rat intestinal epithelial cells (IEC-6), where GR activation suppresses FXR downstream activity [60]. Thus, FXR may promote downstream transcription of GR, while also experiencing feedback inhibition by glucocorticoids. Perhaps the most interesting of these findings was the mechanism through which this occurs. Dexamethasone decreased FXR mRNA but even more substantially decreased downstream SHP and FGF15 expression. The authors conclude that dexamethasone is suppressing both FXR and its downstream signals independently via an unknown mechanism. GR siRNA knockdown experiments yielded an increased in SHP and CSE expression at baseline in these cells [60].

FXR and GR pathways may also interact in the upregulation of PPARα in response to glucocorticoid stimulation. In mice, dexamethasone upregulates PPARα activity [60], which, as previously discussed, will decrease the total bile acid pool and decrease FXR activity [50]. Dexamethasone treatment in combination with NSAIDs leads to an increase in TMCA, an FXR antagonist, and a decrease in TCDCA, an FXR agonist [60]. These mice showed worse intestinal enteropathy, presumably from synergistic receptor effects [60]. Interestingly, these animals also showed decreased expression of ileal FXR, which was only seen with combination therapy as neither dexamethasone nor NSAID alone showed such an effect. The combination treatment also led to a substantial decrease in the expression of CSE, a known target of FXR and an important effector for maintenance of intestinal barrier function [46,60] (Figure 2C).

It appears that normal FXR activity is reliant upon the function of the GR, as there is diminished FXR function in the setting of GR KO mice. Similarly, functional FXR is important for GR activity given the FXR response element in the GR promoter. This is further confirmed with the decreased activity of GR receptor in FXR KO mice, and the relative decreased response of FXR KO mice to dexamethasone treatment. While FXR does not function normally in the absence of the GC receptor, robust activation of the GC receptor itself seems to dampen the expected activity of FXR. This most likely occurs through the pathway of increased PPARa-UGT leading to decreased total BA pool and consequently decreased FXR activity; however, that does not fully explain the diminished activity of FXR in GC receptor KO models. Thus, further work elucidating this relationship is required. FXRs interaction with GR is summarized in Figure 2.

The COX pathway is also well known for its role in gut inflammation and manipulation of this pathway via NSAIDs are known to cause GI injury. FXR may be involved in this action. In fact, relative to WT, FXR KO mice experience more severe damage of the gastric mucosa when treated NSAIDs and have increased expression of myeloperoxidase, TNFα, and inducible nitric oxide. This was in addition to reduced expression of CSE and endothelial nitric oxide. In WT mice, histologic injury and intestinal bleeding were attenuated by treatment with GW4064, a synthetic FXR agonist, prior to NSAID exposure [45]. These findings suggest an interaction between FXR activity and gut anti-inflammatory pathways and opens the door for FXR receptor manipulation in a clinical setting.

### 2.4. Clinical Implications

Given these data, there is interest in manipulating FXR pathways in intestinal inflammatory diseases in humans. In fact, the FXR agonist obeticholic acid (OCA) is FDA approved for use in the liver diseases primary biliary cirrhosis and non-alcoholic steatohepatitis [14]. There exists a potential for therapeutics targeting FXR within the intestine as well. In healthy individuals there exists a proximal-to-distal gradient of FXR expression within the small bowel, with stronger FXR expression distally that reverses in the colon. There is approximately 90% expression in terminal ileum, 64% in the right colon, and 34% in the left colon. However, in patients with microscopic colitis, there is significantly lower expression of FXR in the right and left colon and the proximal-to-distal expression gradient is lost [61]. Evidence suggests that FXR activation improves colitis and inhibits pro-inflammatory cytokine changes in humans [62].

Just as in experimental models, the data regarding changes to FXR expression in humans is not straightforward. FXR mRNA is significantly increased in the ileum, but not the colon, of IBS patients relative to controls [47]. However, others have reported no difference in ileal FXR between control samples and those from Crohn’s disease (CD) and ulcerative colitis (UC) patients [62]. They did find a decrease in expression of downstream SHP in ileal samples from patients with CD, but changes in patients with UC were not significant, perhaps representing a difference in the pathophysiology of CD and UC [62]. A similar decrease was also seen in FGF19 expression in adult patients with CD [57,63]. Interestingly, children with either UC or CD have significantly decreased expression of FXR in areas of active disease [64], which may explain why childhood IBD responds differently than in adults.

FGF19 levels shows promise as a biomarker for assessing FXR and microbiome changes. In the pathophysiology of Clostridium difficile colitis (C. diff), fecal microbiota transplant (FMT) is a well-established treatment. The resulting change in microbial composition can lead to alterations in the bile acid pool, which alters FXR activation. Indeed, FMT is associated with an increase in FGF19 signaling suggesting increased FXR activity [65]. Alteration of the bile acid pool with UDCA treatment yields similar results in murine C. diff models, and UDCA administration leads to increased expression of the FXR/FGF15/19 pathway in both cecal and colonic tissue, presumably through its microbiome effects [66].

Given the heterogeneity of FXR at the genomic level [10], genetic variability in FXR may lead to important disease-specific effects. Currently, single-nucleotide polymorphisms in the NR1H4 (FXR) gene are significantly associated with CD and UC [67]. Additionally, certain FXR minor alleles predict overall surgery risk and timing of progression to surgery in women with CD. Some cell models suggest that this may be due to Estrogen receptor effects on FXR activity [68]. While these data provide an exciting framework for future research, a clearer understanding of the function of FXR is needed before utilizing them in clinical disease. The clinical roles of FXR within intestinal diseases is summarized in Table 2.

## 3. Barrier Function

Maintenance of the intestinal barrier is critical for both mechanical protection and gut homeostasis. Disruption of this barrier leads to protein loss, electrolyte imbalances, bacterial translocation, gut-origin sepsis, and death. Data suggest that FXR plays an important role in barrier homeostasis, both in normal physiology and in response to pathophysiologic insult.

### 3.1. Histology

FXR activation in the setting of a pathologic insult can prevent deleterious histological intestine injury observed in multiple models of barrier injury, including LPS [69,70], ischemia-reperfusion injury (IRI), [43,44], DSS colitis [5], and trinitrobenzensulfonic acid (TNBS) [5]. In LPS-induced injury, FXR activation via taurodeoxycholic acid (TDCA), a conjugated bile acid and natural FXR ligand, prevents intestinal mucosal injury while maintaining villus height and architecture, returning histology to control levels. This same histological rescue with TDCA treatment was not observed in LPS-treated FXR KO mice [69]. Importantly, FXR KO mice do not appear to have baseline intestinal architectural differences; however, they exhibit more villus necrosis and inflammatory cell infiltration following LPS treatment [70]. Treatment with the FXR agonist GW4064 also prevents villus necrosis and inflammatory cell infiltrate in LPS-induced injury [70]. In IRI models, pre-treatment with the FXR agonist OCA prevents decreases in villus length, decreases in trans-epithelial electrical resistance (TEER), and increases in Park/Chiu scores, a measure of barrier injury in ischemia [43,71,72]. Here, FXR KO mice demonstrate a worse Park/Chiu score and substantially worse survival relative to WT (38% vs. 62%) [44]. These data suggest that FXR activation is favorable to the intestinal architecture in acute injury.

### 3.2. Functional and Homeostatic Effects

While histologic changes in the intestine are important, breakdown of barrier integrity and function leads to sepsis. FXR activation shows promise for ameliorating such dysfunction. In fact, FXR activation has been shown to decrease bacterial translocation, albumin loss, endo-toxin translocation, and FITC-dextran leakage [42,43]. In mice subjected to LPS injury, there is an increase in FITC leakage and endotoxin within the bloodstream; however, FXR activation via CDCA abolished the serum increases of both [73]. In IRI, FXR activation via OCA, blunts decreases in TEER, a measure of barrier integrity [43]. In murine cirrhosis, FXR activators OCA and Fexeramine both decrease bacterial translocation and albumin loss [42,74]. OCA treatment also leads to partial restoration of epithelial goblet cells that are typically lost in murine cirrhosis [74].

As seen previously, there are data that conflict with these findings. For example, in Caco2 cell models of epithelial barrier function, FXR activation via CDCA decreased TEER, increased permeability, and increased IL8 release, which is opposite to what was described above. Additionally, FXR inhibition with Z-guggulsterone did not reverse CDCA-induced decreases in TEER [47], calling this described mechanism into question. It may also speak to the affinity of CDCA for FXR relative to Z-guggulsterone. Additionally, unexpected findings with FXR activation have been observed in T84 polarized monolayers. Here, FXR activation via both DCA and GW4064 impaired wound closure and decreased cell migration. Interestingly, in HEK289 cells, this same group found that FXR activity may inhibit expression of apical cystic fibrosis trans-membrane conductance regulator Cl^−^ channels (CFTR), as activation via both DCA and GW4064 decreased total and surface CFTR expression. Activation did not affect expression of other components of the Cl^−^ secretory pathway in colon. It appears that this inhibition of CFTR occurs at the level of transcription by inhibiting CFTR promoter activity, although the mechanism of FXR interaction is not fully delineated. Interestingly, in T84 monolayers, CFTR activity was required for colonic epithelial restitution, similar to the mechanism seen in airway epithelial cells [75], so perhaps FXR indirectly inhibits barrier repair. These seemingly contradictory findings make it difficult to draw definitive conclusions on the role of FXR.

### 3.3. Tight Junctional Proteins

Tight junctional proteins (TJP), including zona occludens (ZO), claudins, and occludin, play an important role in the maintenance of intestinal barrier integrity, and their function appears to be linked to FXR activity. FXR activation in the intestine may alter TJP architecture. In murine models of cirrhosis, there is a consistent decrease in TJP expression [42,76] that is further diminished with FXR activation. In bile duct ligation (BDL), a model of cholestasis and cirrhosis, barrier integrity is restored in ileum of rats by treatment with OCA via increased expression of TJPs claudin-1 and occludin [76]. In another cirrhosis model, OCA leads to an increase in expression of ZO-1 and occludin [42]. OCA treatment in cirrhosis also increases expression of claudin-1 [74].

FXR KO mice seem to have a lower baseline expression of TJPs ZO-1 and claudin-1 relative to WT. In LPS-induced injury, an overall decrease in the expression of TJPs is observed in WT mice and a more substantial decrease in FXR KO mice. Treatment with GW4064 blunts the decreases in ZO-1 and claudin-1 expression only in WT animals [70]. CDCA, the strongest endogenous FXR ligand, also prevents LPS-induced decreases in ZO-1, occludin, and claudin-1. Epithelial myosin light chain kinase (MLCK) is known to play an important role in TJP regulation, and increased MLCK activity is recognized as deleterious for TJP function [77]. LPS injury in mice leads to an increase in MLCK activity, and FXR activation via CDCA suppresses this MLCK increase. In an IPEC-2 cell model of intestinal barrier, CDCA does not lead to MLCK inhibition when FXR is knocked down, suggesting these changes are FXR-dependent. Importantly, IPEC-2 cells do demonstrate MLCK inhibition with FXR agonism. In the presence of an MLCK inhibitor, a similar prevention of LPS-induced TJP damage was observed, seeming to confirm that FXR activation leads to MLCK inhibition [73].

**Figure 3:**
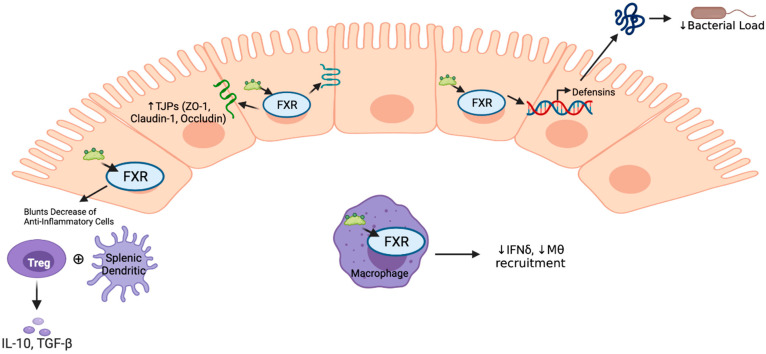
FXR plays numerous roles in maintaining barrier integrity and promoting decreased inflammation. TJP—tight junction proteins; Treg—t regulatory cells; IL—interleukin; ZO—zona occludens; Mθ—macrophage (created with BioRender.com accessed on 10 October 2021).

Downstream activation of the FXR pathway may also impact TJP function. Mice with DSS colitis typically show decreased TJP function; however, treatment with the FGF analogue M52 leads to claudin and occluding induction. This was not seen in FXR KO mice treated with M52, suggesting FXR dependence. Additionally, M52 treatment increased expression of transcription factors important for enterocyte function [57]. These FXR-dependent changes in TJP may help explain the barrier protection seen in some models with FXR treatment. These findings are summarized in Figure 3.

### 3.4. Proliferative Effects

The intestinal barrier is also influenced by cell proliferation, and FXR, acting at the transcription level, has been observed to promote homeostatic activity and function of regulatory elements, including c-Myc [69]. Cellular stress diminishes proliferative cellular activities. Yet, in LPS injury models, FXR activation via TDCA has been shown to maintain mucosal proliferation and increase the number of epithelial cells in S-phase, effects not observed in TDCA-treated FXR KO mice. In IEC-6 cells, TDCA led to an increase in the total number of cells relative to untreated controls via a c-Myc-dependent pathway as TDCA treatment lead to increases in both FXR and c-Myc activity. These findings were not seen in FXR siRNA knockdown cells [69]. The idea that FXR is vital for barrier maintenance is further suggested by its dysregulated role in epithelial cancer models, where it has been shown that FXR antagonism leads to a dose-dependent increase in malignant cell proliferation. In human colon cancer cell lines, FXR activation via GW4064 leads to a dose-dependent attenuation of cellular proliferation. In nude mice with human cancer xenografts, reductions in tumor weight and volume were observed with FXR activation. The underlying basis of this response seems to be an FXR-mediated inhibition of EGFR, Src, and ERK1/2 phosphorylation. Interestingly, FXR inhibition via Z-guggulsterone increased EGFR activity [78]. Others have also demonstrated an anti-proliferative role for FXR, where FXR activation with DCA was shown to inhibit activity of the EGFR/Src/ERK pathway in IEC-6 cells. Inhibiting FXR with Z-guggulsterone or siRNA knockdown eliminated the anti-proliferative effects [79]. The role of FXR in proliferation and tumorigenesis is most well described in relationship to hepatocellular carcinoma in the liver, and these concepts provide a helpful framework by which to examine the role of FXR in GI malignancy.

Further evidence for the role of FXR in barrier homeostasis is observed in interactions with the adenomatous polyposis coli (APC) tumor suppressor gene, as inactivating mutations in APC genes lead to a decreased expression of FXR, mediated by caudal homeobox-2 (CDX2). Intestinal FXR expression may be proportional to the allelic quantity of CDX2 within stem cells, and evidence suggests that CDX2 may be required to induce FXR expression during enterocyte differentiation [80]. It has been observed that FXR KO mice are significantly more susceptible to intestinal tumorigenesis than their WT counterparts, and when mice lack both FXR and APC, a synergistic effect on tumorigenesis is observed [54].

Building on this work in APC-deficient mice, treatment with T-BMCA, a murine-specific endogenous FXR antagonist, leads to a marked increase in tumor proliferation in murine ileum. Interestingly, over a longer term, T-BMCA decreased intestinal integrity, yielded higher permeability, and accelerated tumor growth in both small bowel and colon. In these mice, T-BMCA treatment also increased IFNγ, IL6, and IL17, similar to mice maintained on a pro-inflammatory diet. T-BMCA-treated mice also show downregulation of FXR target genes. In enteroid culture from APC-deficient mice, fexeramine blocked T-BMCA-induced proliferation. FXR agonism also inhibited cancerous organoid growth, downregulated uncontrolled stem cell proliferation, and stimulated tumor suppressors. Conversely, inhibition of FXR in colonic stem cells resulted in uncontrolled proliferation and adenoma-to-adenocarcinoma progression. Deletion of FXR from enteroids increased growth and new organoid formation. FXR also seems to impact chromosomal stability, as FXR inhibition led to increased DNA double-stranded breaks in this model [81]. Taken together, these data suggest that stimulating FXR inhibits uncontrolled proliferation in tumor models and helps to maintain function of tumor suppressors (Table 2).

## 4. Immune Response

The immune system within the GI tract serves an important role in mitigating pathophysiologic insults and is another area where FXR may play a key role. FXR interacts with the immune system through expression within both the epithelium and the immune cells themselves [55]. Early work has indicated that FXR activation prevents bacterial overgrowth within the intestinal lumen [82], and intestinal bacteria are a key driver of the gut immune system [83]. One mechanism through which this occurs is via FXR transcriptional regulation of anti-microbial peptides. In murine BDL, OCA increases expression of these peptides and decreases the bacterial load within the intestinal lumen [42].

Toll-like receptors (TLRs) are important in the gut response to microbes, and their signaling is interconnected with FXR activity [84]. In human CD14-derived peripheral blood mononuclear cells (PMBCs), activation of some extracellular TLRs down-regulates FXR expression, whereas activation of TLR9 induces FXR. Similarly, in murine splenic-derived monocytes and Raw264.7 macrophages, TLR9 response elements function as FXR promoters. Conversely, when PMBCs lack TLR9, FXR expression is substantially decreased, a finding also observed in vivo with TLR9 KO mice. FXR seems to be the main driver, as activation provides some protection from TNBS colitis in MyD88/TLR9 KO mice. The same is not true in FXR KO mice with normal TLR9, as they experience worse injury than WT. Therefore, while FXR expression and activity are closely linked to TLR function, FXR activity is not wholly dependent upon TLRs [85]. Similar findings are observed in TLRs that negatively regulate FXR, such as TLR4. In the setting of LPS injury, TLR4 expression is increased; however, this can be blocked by GW4064. This negative regulation is further observed in FXR KO mice, which have increased TLR4 expression relative to WT mice [70]. The same is demonstrated in murine cirrhosis, where TLR4 expression is increased. With FXR activation, TLR4 expression returns to control levels [42]. These data suggest a consistent interaction between FXR and TLRs, with individual subtypes either increasing or decreasing FXR activity; however, FXR activity is not wholly dependent on TLR activity.

FXR also plays a role in the balance of immune cell recruitment. In BDL-induced inflammation, OCA decreases ileal recruitment of immune cells including macrophages, T cells, dendritic cells, and B cells. A similar decrease was observed in both splenic and mesenteric lymph nodes. A resultant decrease in local expression of IFNγ in the ileum was observed secondary to decreased macrophage recruitment and macrophage IFNγ signaling [76]. Others have confirmed the role of FXR in limiting immune cell recruitment. In LPS-induced ileal injury, FXR activation stimulated macrophage recruitment within the lamina propria. Importantly, FXR KO mice have more macrophages present in the ileum at baseline versus WT [70]. As mentioned earlier, FXR activation limits transcription of proteins that promote autophagic influx [43]. Treatment with FGF15 analogues decreases macrophage recruitment, but is not functional without FXR present [57].

FXR activity in immune cells impacts their functional response to insult. In Raw264.7 macrophages, FXR activation leads to down-regulation of IFNγ signaling [86], and, not surprisingly, FXR KO macrophage have higher baseline IFNγ expression than WT [56]. Conversely, when macrophages are pre-treated with IFNγ, FXR expression is decreased. This may be due to IFNγ inducing negative transcription factors of FXR, including STAT1 [86]. In DSS colitis, FXR activation prevents decreases in more anti-inflammatory splenic dendritic cells and T-regulatory cells, while inhibiting increases in pro-inflammatory chemokines like Madcam1 in pro-inflammatory cells. Inhibiting this Madcam1 interaction with T cells is known to ameliorate symptoms of colitis. FXR activation also increases the expression of chemokines, which attract anti-inflammatory T-regulatory cells [41]. While complex and still worthy of much investigation, it seems that FXR activation promotes a more anti-inflammatory immune response in the setting of intestinal injury and provides protection from pathogenic insults (Figure 3).

## 5. Conclusions

Current evidence suggests that FXR modulates the inflammatory response, improves barrier function, limits tumorigenesis, and alters the innate immune response within the GI tract. FXR manipulation many have a role in the treatment of chronic disease, as in the liver, and a role in acute critical illness, bolstering barrier function and dampening inflammation to prevent GI dysfunction. Further defining FXR’s relationship to inflammation, barrier function, immune modulation and tumorigenesis will allow improved therapeutic manipulation of FXR signaling in clinical applications [13,14,15,16,33]. However, the conflicting results, due in part to poor FXR specificity in existing agonists/antagonists [70,87], must be rectified prior to direct clinical applications. It is, however, clear that FXR plays a vital role in intestinal physiology and pathophysiology.

## Figures and Tables

**Table 1:** Known FXR responses in experimental models of inflammation (SHP—small heterodimer protein; IL—interleukin; PPARα-UGT—peroxisome proliferator-activated receptor (PPARα)-UDP-glucuronosyltransferases (UGTs); Veg—vascular endothelial growth factor; FGF—fibroblast growth factor, IFN—interferon; madcam—mucosal addresin cell adhesion molecule).

Experimental Conditions	Intervention + Observed Effects
DSS Colitis	FXR Activation: ↑ IL10, SHP, FGF15, ↓IL1β ↑PPARα-UGT: ↓ FXR activity +worse injury PPARα-UGT KO mice: ↑FXR activity, ↓Injury Treatment with FGF15 analogue: ↓ IL1β, IL6, TNFα No effect in FXR KO mice
Ischemia Reperfusion Injury	FXR Activation Blunts increase Lactate, LDH, IL6, IL1β, IFNγ, Limits autophagic influx
Cirrhotic Rats	FXR Activation ↓ IFNγ, TNFα, MadCam-1, IL17, IL10
O/G Deprivation	FXR Activation ↑cell viability, ↓NFκB, TNFα, IL6
CDCA treatment in CACO-2	FXR Activation ↑IL8 IL6, TNFα, VegF FXR Inhibition Blunts Increases in IL8, IL6
High Fat Diet in Fish	↓FXR activity leads to increase in pro-inflammatory signaling CDCA dietary supplementation ↓IL1β, TNFα, COX-2, IL6 ↑ IL10

**Table 2:** Described roles of FXR in known clinical diseases of the intestine (CD—Crohn’s disease, FMT—fecal microbiota transplant).

Disease Process	Proposed FXR Role
Microscopic Colitis	Loss of normal proximal-to-distal FXR expression gradient
Crohn’s Disease	No observed difference in FXR expression relative to healthy control Decreased ileal SHP and FGF19 Minor alleles within FXR gene → Increased surgery risk? SNP at FXR gene (NR1H4) → Increased Risk of CD
Pediatric Crohn’s and Ulcerative Colitis	Decreased FXR expression
C. difficile colitis	FMT leads to increased FGF-19 expression (FMT→ ↑total bile acids? → ↑ FXR activity? → ↑FGF-19)
Malignancy	APC inactivating mutations: decreased FXR expression Decreased FXR mRNA expression in colitis associated neoplasia Decreased FXR expression CRC and pre-malignant polyps
